# Improvement of Renal Function by Long-Term Sustained Eculizumab Treatment in a Patient with Paroxysmal Nocturnal Hemoglobinuria

**DOI:** 10.1155/2015/673195

**Published:** 2015-06-01

**Authors:** Haruhiko Ninomiya, Naoshi Obara, Akiko Niiori-Onishi, Yasuhisa Yokoyama, Mamiko Sakata-Yanagimoto, Yuichi Hasegawa, Shigeru Chiba

**Affiliations:** ^1^Department of Medical Sciences, Faculty of Medicine, University of Tsukuba, Tsukuba 305-8575, Japan; ^2^Department of Hematology, Faculty of Medicine, University of Tsukuba, Tsukuba 305-8575, Japan; ^3^Department of Internal Medicine, Hitachi, Ltd., Hitachinaka General Hospital, Hitachinaka 312-0057, Japan

## Abstract

Chronic kidney disease (CKD) is one of the major manifestations of paroxysmal nocturnal hemoglobinuria (PNH). CKD in PNH is induced mainly by intravascular hemolysis of PNH-affected red blood cells (RBC) missing the glycosylphosphatidylinositol-anchored proteins with complement-regulatory activities, CD55 and CD59. CKD develops by heme absorption in the proximal tubules resulting in the interstitial deposition of iron in the kidneys. We administered eculizumab to a patient with PNH, who was one of 29 patients enrolled in the AEGIS clinical trial, an open-label study of eculizumab in Japan. The patient was complicated by stage 3 CKD with impaired estimated glomerular filtration rate (eGFR), at grade G3b, and had obvious proteinuria (2-3+, 1-2 g/day). In a two-year extension to the 12-week AEGIS study, eGFR improved significantly, and the eGFR has since been maintained at grade G2 without proteinuria by sustained eculizumab treatment (>6 years). Renal function improved and maintained by long-term sustained eculizumab treatment, presumably by clearance of iron from the kidney as well as inhibition of the production of anaphylatoxin C5a, even in advanced stages of CKD, is one of the benefits of eculizumab treatment in PNH.

## 1. Introduction

Renal failure is one of the important factors which determine the prognosis of paroxysmal nocturnal hemoglobinuria (PNH), especially for Japanese patients. It has been reported that renal failure is responsible for 18% of deaths and is a significant risk factor for Japanese PNH patients [1]. Renal dysfunction in PNH is believed to be related to microvascular thrombosis in the glomeruli and hemoglobinuria, resulting in the interstitial deposition of iron in the kidneys [2].

The first clinical investigation of eculizumab was an open-label, 12-week, Phase II pilot study that enrolled 11 patients in two UK study centers [3]. Hillmen et al. reported that 64% of PNH patients showed evidence of chronic kidney disease (CKD) [4]. They reported that long-term sustained eculizumab treatment resulted in a time-dependent improvement of renal function in PNH; 93.1% of patients exhibited improvement (44.8%) or stabilization (48.3%) in CKD score at 36 months [5]. Improvement in renal function was more commonly seen in patients with baseline stages 1-2 CKD, although improvement was also observed in patients with stages 3–5 CKD [4]. Long-term observation of Japanese PNH patients in a two-year extension to the 12-week AEGIS study also showed that the majority of patients exhibited stable (56%) or improved (41%) renal function [6].

Here, we report a long-term (>6 years) observation of a PNH patient, with baseline stage 3 CKD, who was enrolled in the AEGIS clinical trial [7], and demonstrated the maintenance effect of eculizumab on renal function.

## 2. Case Report

A 70-year-old male PNH patient was enrolled in an open-label study of eculizumab, as one of 29 patients enrolled in the AEGIS clinical trial in Japan [7]. The trial was sponsored by Alexion Pharmaceuticals. His disease was diagnosed on admission to a regional hospital due to urinary tract infection, two years before enrollment in the study. At the time of diagnosis, laboratory data indicated pancytopenia, leukocytes 3 × 10^9^/L, hemoglobin (Hb) 7.7 g/dL, platelet 99 × 10^9^/L, total bilirubin 2.7 mg/dL, and 886 U/L LDH. A chromosomal analysis of bone marrow cells revealed an abnormality, 46 XY, t(1;7) (q44;q11), in 4 of 20 cells. A flow cytometric study of peripheral blood revealed significant ratios of PNH-affected, CD59-deficient, red blood cells (RBC) (29.5%), neutrophils (86.5%), and monocytes (88.9%). A diagnosis of PNH with myelodysplastic syndrome was made. Before enrollment in the AEGIS clinical trial, constant RBC transfusions (2–4 U/month) had been required. No thromboembolic episodes had been demonstrated. Before the diagnosis of PNH, he had an almost ten-year past history of diabetes mellitus (DM), which had been well controlled by insulin therapy (glycoalbumin 18–21%).

Administration of eculizumab (initiated on February 12, 2008) resulted in rapid reduction of LDH levels, and he became RBC transfusion-independent, indicating efficient inhibition of intravascular hemolysis by eculizumab. He was hospitalized twice (January to February, 2009, and November to December, 2010) due to hyperbilirubinemia, due to uncertain causes. These serious adverse events (AE) were assessed as unrelated to the eculizumab treatment, because continuation of eculizumab administration did not worsen the AE. No obstructive biliary tract lesions or known hepatic virus infections were detected by laboratory examinations, and he recovered from the AE spontaneously on both occasions.

**Figure 1:**
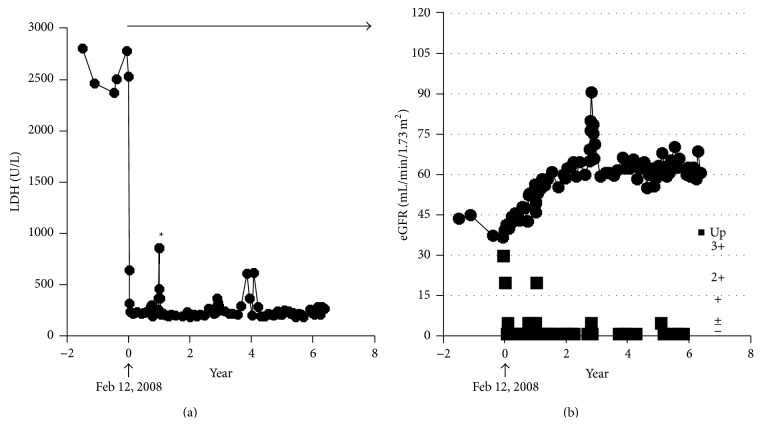
Clinical course of the patient. On February 12, 2008, he was enrolled in the AEGIS clinical trial. (a) Administration of eculizumab resulted in a rapid reduction of LDH. Asterisk indicates the increased LDH due to delayed administration of eculizumab during an AE of hyperbilirubinemia. Arrow indicates eculizumab treatment. (b) He was complicated with stage 3 CKD, grade G3b, and obvious proteinuria, at baseline. Eculizumab treatment improved eGFR gradually. Since two years after introduction of eculizumab, eGFR at grade G2 and no proteinuria have been maintained. Closed squares denote grades of proteinuria (−, ±, +, 2+, and 3+).

**Figure 2:**
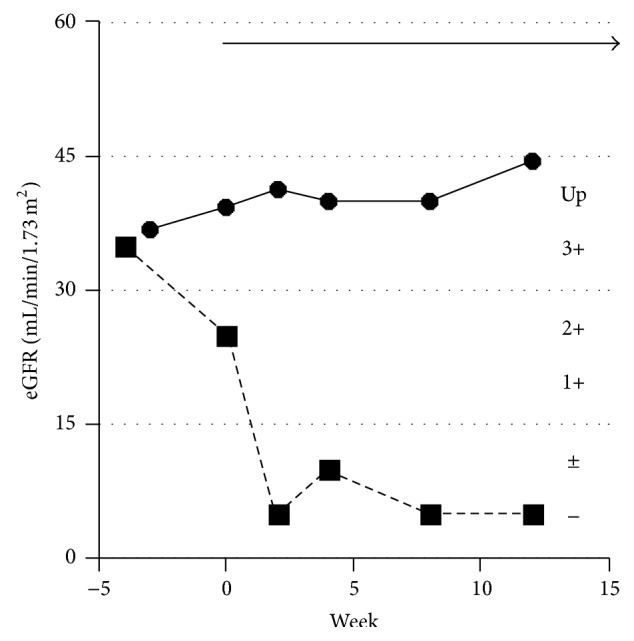
eGFR and proteinuria around the initiation of eculizumab. Obvious proteinuria (closed squares, up), and quickly disappearing proteinuria by initiation of eculizumab treatment (arrow). Closed circles denote eGFR.

As shown in Figure 1, at the time of enrollment in the AEGIS clinical trial, an impaired estimated glomerular filtration rate (eGFR) with obvious proteinuria (1-2 g/day), grade G3b (eGFR 30–45 mL/min/1.73 m^2^) according to the modified 2012 KDIGO CKD guideline adopted for the Japanese [8], was seen. In a two-year extension to the 12-week AEGIS study, renal function gradually improved to stage 2 CKD, grade G2 (eGFR 60–90 mL/min/1.73 m^2^), without proteinuria, which was maintained by sustained eculizumab treatment. Proteinuria disappeared soon after the initiation of eculizumab (Figure 2). Improved eGFR levels and no proteinuria have been maintained throughout the 6-year eculizumab treatment (until the patient was 77 years old). Proximal tubular dysfunction was not apparent by the urinary level of liver-type fatty acid binding protein (2.5 *μ*g/g·Cr). Serum ferritin levels were increased both before (459 ng/mL) and after six years of treatment with eculizumab (547 ng/mL, June 2014). Because the patient's DM had been well controlled by insulin therapy, this improvement in renal function seems to have been induced by the long-term administration of eculizumab, which inhibited intravascular hemolysis and hemoglobinuria (renal hemosiderosis).

## 3. Discussion

Chronic intravascular hemolysis results in iron deposition in the kidney in almost all PNH patients. This excess iron can be detected by magnetic resonance imaging [9, 10]. Excess iron deposition can result in proximal tubular dysfunction [2, 11, 12]. Chronic renal failure due to hemosiderosis and subsequent interstitial scarring may occur in patients with long-standing PNH [2, 13]. Inhibition of production of anaphylatoxin C5a by eculizumab is another possible mechanism improving the glomerular function of PNH, as reported in cases of dense deposit disease and C3 glomerulonephritis [14].

Improvement of renal function in PNH by administration of eculizumab is time-dependent, as reported by Hillmen et al. [5] and Kanakura et al. [6]. Improvement of renal function is less common in the advanced stages 3–5 of CKD than stages 1-2 of CKD, even with long-term administration of eculizumab, whereas patients with stages 3–5 did not worsen during treatment with eculizumab [4]. Improvement of renal function in our case may be partly explained due to the initiation of eculizumab treatment relatively soon after the onset of PNH. At the time of enrollment in the AEGIS clinical trial, the ratio of PNH-affected RBC was <30%, which suggests a relatively small amount of iron may have been deposited in the kidneys. The antithrombotic effects and anticomplement activity (inhibition of C5a production) of eculizumab also contributed to the improvement of glomerular function, because obvious proteinuria disappeared soon after administration (Figure 2).

Our case shows the clinical effects of eculizumab on the improvement of renal function even in patients with advanced stage CKD. When eculizumab treatment is introduced at an early stage of PNH, prognosis may be improved by recovery of renal function.
